# The mechanistic and prognostic implications of heart rate variability analysis in patients with cirrhosis

**DOI:** 10.14814/phy2.15261

**Published:** 2022-04-19

**Authors:** Noor‐Ul‐Hoda Abid, Ali R. Mani

**Affiliations:** ^1^ 4919 Network Physiology Lab Division of Medicine UCL London UK; ^2^ Lancaster Medical School Lancaster University Lancaster UK

**Keywords:** autonomic dysfunction, cirrhosis, heart rate variability, liver, MELD, network physiology, survival

## Abstract

Chronic liver damage leads to scarring of the liver tissue and ultimately a systemic illness known as cirrhosis. Patients with cirrhosis exhibit multi‐organ dysfunction and high mortality. Reduced heart rate variability (HRV) is a hallmark of cirrhosis, reflecting a state of defective cardiovascular control and physiological network disruption. Several lines of evidence have revealed that decreased HRV holds prognostic information and can predict survival of patients independent of the severity of liver disease. Thus, the aim of this review is to shed light on the mechanistic and prognostic implications of HRV analysis in patients with cirrhosis. Notably, several studies have extensively highlighted the critical role systemic inflammation elicits in conferring the reduction in patients’ HRV. It appears that IL‐6 is likely to play a central mechanistic role, whereby its levels also correlate with manifestations, such as autonomic neuropathy and hence the partial uncoupling of the cardiac pacemaker from autonomic control. Reduced HRV has also been reported to be highly correlated with the severity of hepatic encephalopathy, potentially through systemic inflammation affecting specific brain regions, involved in both cognitive function and autonomic regulation. In general, the prognostic ability of HRV analysis holds immense potential in improving survival rates for patients with cirrhosis, as it may indeed be added to current prognostic indicators, to ultimately increase the accuracy of selecting the recipient most in need of liver transplantation. However, a network physiology approach in the future is critical to delineate the exact mechanistic basis by which decreased HRV confers poor prognosis.

## Graphical abstract

Several lines of evidence have revealed that decreased heart rate variability holds prognostic information and can predict survival of patients independent of the severity of liver disease. This article reviews the mechanistic and prognostic implications of Heart rate variability analysis in patients with cirrhosis.

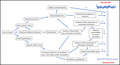

## 1 INTRODUCTION

Globally, 1 million patients approximately die from cirrhosis every year (Asrani et al., 2019; Mokdad et al., 2014). Cirrhosis, the end result of chronic inflammation and fibrosis, confers the defective liver function, as diffuse scar tissue and regenerative nodules displace functional hepatocellular tissue. Consequently, affecting the function of several other organ systems, due to the critical role the liver elicits, which thus results in a number of extra‐hepatic manifestations including ascites, hepatic encephalopathy, as well as hepatorenal syndrome and also defective immune function. All of which account for significant morbidity in this patient population and subsequently, a poor prognosis which often proves fatal (Dong & Karvellas, 2019; Møller & Bendtsen, 2015), ultimately resulting in the need for liver transplantation.

Due to the multisystem involvement in cirrhosis, a network physiology approach has been suggested for the optimum assessment of patients suffering from chronic liver failure (Tan et al., 2020; Zhang et al., 2022). Crucially, a network physiology approach may help develop physio‐markers to create novel prognostic indicators for patients with cirrhosis, which has been the subject of investigation by recent studies (Bhogal et al., 2019; Bottaro et al., 2020; Chan et al., 2017; Jansen et al., 2018, 2019; Mani et al., 2009; Satti et al., 2019). Currently, most physio‐markers such as heart rate variability (HRV) or temperature variability indices reflect a complex ‘functional’ interaction between different physiological regulatory systems. Thus, highlighting that they may indeed provide insight into the pathophysiology and hence prognosis of multi‐systemic disorders such as sepsis and liver cirrhosis.

HRV has been used extensively to delineate the prognosis of patients suffering from a variety of conditions due to its non‐invasive use (Ahmad et al., 2009; Braunisch et al., 2020; Poliwczak et al., 2019; Yamada et al., 2020). Notably, decreased HRV is known to be indicative of poor prognosis in patients with cirrhosis and can predict survival of patients independently to MELD, the Model for End Stage Liver Disease currently used in the clinic to assess the severity of liver dysfunction in patients on the liver transplant list (Bhogal et al., 2019; Oyelade et al., 2020; Satti et al., 2019). Furthermore, the use of HRV concomitant with MELD holds the potential to increase the accuracy of prognostication and hence in identifying those most in need for liver transplantation.

Fundamentally, the variability depicted in the normal cardiac sinus rhythm is the result of a multifaceted relationship between the cardiac sinoatrial node (SAN) pacemaker and the autonomic nervous system (Goldberger et al., 2002). Moreover, decreased HRV is reflective of an increase in the extent of regularity exhibited in the cardiac rhythm, which is postulated to be as a consequence of uncoupling occurring between the individual constituents giving rise to the normal variability exhibited in the cardiac rhythm. Thus, uncoupling between the afferent inputs, namely the autonomic nervous system and the cardiac pacemaker (Buchman, 2002; Pincus, 1994). However, a variety of different mechanistic hypotheses have also been suggested to explain the occurrence of decreased HRV, which will be delved on further in this review, to ultimately shed light on the mechanistic and prognostic implications of HRV analysis in patients with cirrhosis.

## 2 HEART RATE VARIABILITY IN CIRRHOSIS

**FIGURE 1:**
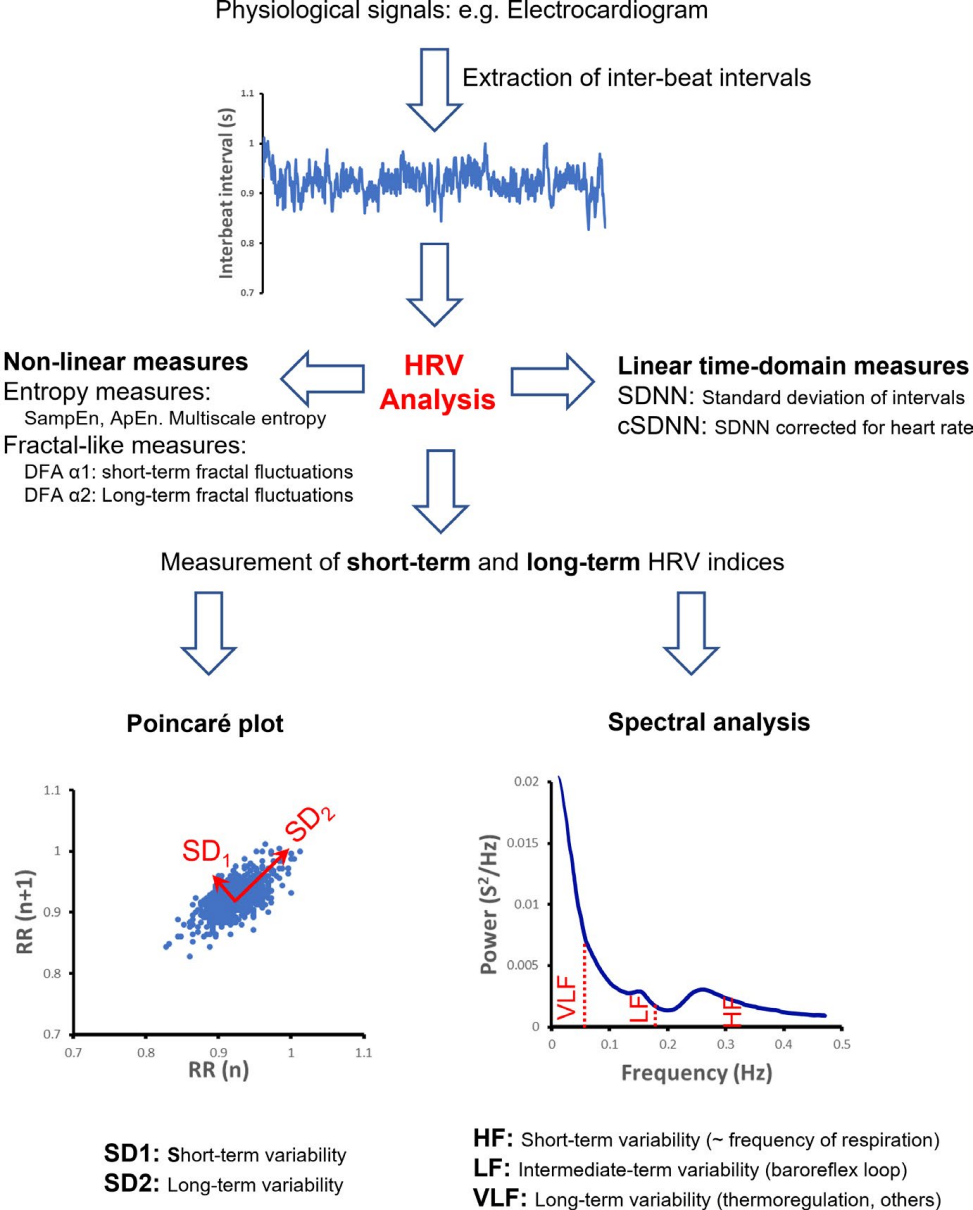
An overview of heart rate variability (HRV) analysis. Various physiological signals (e.g., electrocardiogram, plethysmography) can be used for extraction of inter‐beat interval time‐series. Total HRV can be measured by calculation of standard deviation of inter‐beat intervals (SDNN). Recent studies have shown that correction of SDNN for basal heart rate (cSDNN) improves the accuracy of SDNN for assessment of autonomic function. Short‐term and long‐term HRV can be calculated using a variety of methods such as spectral analysis and Poincare’ plot. Non‐linear indices assess the pattern of heart rate fluctuations such as irregularity (e.g., entropy) and fractal‐like fluctuations. SampEn: Sample Entropy, ApEn: Approximate Entropy, DFA: Detrended Fluctuation Analysis, HF: High Frequency, LF: Low Frequency, VLF: Very Low Frequency. A detailed explanation of this figure is outlined further in Appendix 1

There are several methods for the measurement of HRV (see Appendix 1 and Figure 1 for more information). Some of these methods describe the total extent of HRV within the sinus rhythm, regardless of its pattern. Additionally, other measures quantify the extent of short‐term variability (i.e., respiratory sinus arrythmia) and also long‐term variability (i.e., the baroreflex loop, thermoregulation etc) which give insight into the distinct aspects underpinning the fluctuations exhibited in patients’ HRV. Importantly, these indices can be measured using frequency‐based methods such as spectral analysis or an alternative method known as the Poincare plot analysis (Figure 1) which has frequently been used within the context of cirrhosis (Oyelade et al., 2021; Satti et al., 2019). It is well documented that patients with cirrhosis possess reduced HRV in both their short‐term and long‐term HRV components. Hence, several mechanisms have indeed been proposed for the reduction of HRV in cirrhosis, from systemic inflammation to impaired thermoregulatory dynamics (Bottaro et al., 2020). Thus, the purpose of this section is to describe the mechanisms conferring the reduction in patients’ HRV.

### 2.1 Mechanistic implications

Pivotal studies have revealed that systemic inflammation elicits a crucial role in conferring poorer outcomes in patients with cirrhosis (Clària et al., 2016) and is associated with impaired heart rate dynamics, namely decreased HRV (Bhogal et al., 2019; Haddadian et al., 2013), as well as changes in the pattern of patients’ core body temperature profile (Mani et al., 2018). Importantly, its critical role in eliciting a contributory reduction in patients’ HRV has been extensively highlighted by several studies revealing that the reduction in HRV in patients with cirrhosis correlates with increased cytokine levels (Mani et al., 2009). Mechanistically, it has been proposed that decreased HRV at baseline in patients with cirrhosis could potentially be due to endotoxemia or increased bacterial translocation from patients’ GI tract, which in the context of defective liver function results in systemic inflammation, consequently resulting in decreased HRV (Mani et al., 2009). Crucially, IL‐6 has been found to exhibit a significant inverse correlation with decreased HRV variables, whereby Mani et al., 2009 revealed that increased IL‐6 levels in patients’ blood were inversely proportional to total HRV and long term HRV indices (e.g. SDNN and SD2 respectively). This was also reported in a number of studies in the context of cirrhosis (Bhogal et al., 2019; Mani et al., 2009), as well as in conditions associated with systemic inflammation such as sepsis as well as diabetes mellitus (Aronson et al., 2001; González‐Clemente et al., 2007; Tateishi et al., 2007). In addition, decreased HRV variables are predictive of poor prognosis for patients with sepsis (de Castilho et al., 2018; Goldstein et al., 1998) and cirrhosis (Bhogal et al., 2019; Mani et al., 2009) highlighting that systemic inflammation may indeed be a central mechanism involved in conferring decreased HRV. This is reinforced by the fact that Bhogal et al (2019) revealed that IL‐6 could also predict survival of cirrhotic patients independently to MELD and also crucially affected HRV. Thus, highlighting that decreased HRV is a common feature of several inflammatory disorders, as well as in cirrhosis, whereby both short and long term HRV indices are reduced, but the latter holds key prognostic information for patients with cirrhosis.

Decreased HRV has also been postulated to be due to sympathovagal imbalance as well as vagal neuropathy in various investigations (Ates et al., 2006; Coelho et al., 2001; Hendrickse et al., 1992; Lazzeri et al., 1997, Mani et al., 2009, Miyajima et al., 2001; Newton et al., 2006). Indeed, crucial evidence has been proposed for the development of autonomic neuropathy, such that it has been documented as a frequent complication in approximately 80% of patients with cirrhosis (Bhatti et al., 2021). Consequently, limiting the patient’s ability to appropriately respond to various environmental, as well as physiological stimuli and is thus associated with significant morbidity and poorer outcomes. Mechanistically, this notion of autonomic neuropathy culminating in decreased HRV has been reported to be reflective of uncoupling occurring between the sino‐atrial node (SAN) pacemaker from vagal and thus cholinergic innervation in the context of sepsis and hence profound systemic inflammation (Gholami et al., 2012; Hajiasgharzadeh et al., 2011). This is since Gholami et al., documented that spontaneously beating atria, isolated from rats injected with bacterial endotoxin, exhibit substantially decreased sensitivity to the negative chronotropic effect of cholinergic stimulation (Gholami et al., 2012), which was also demonstrated post IL‐6 injection in mice (Hajiasgharzadeh et al., 2011). This notion of uncoupling was further emphasized by a study emphasizing Pincus’ theory of system isolation (Pincus, 1994), whereby decreased variability concomitant with an increase in the regularity was exhibited in the cardiac cycle of healthy individuals injected with endotoxin (Godin et al., 1996). Likewise, in cirrhotic rats, decreased controllability of the cardiac rhythm was reported post endotoxin challenge, as revealed by an increase in the memory length of the cardiac time‐series (Taghipour et al., 2016). Such a decrease in the controllability has also been reported in patients with cirrhosis (Shirazi et al., 2013), although its clinical significance is not well understood. Collectively, these observations suggest that the cardiovascular system exhibits reduced sensitivity to input from regulatory mechanisms, reflective of partial uncoupling occurring between the autonomic nervous system and the heart.

However, the mechanistic basis by which IL‐6 may potentially be conferring decreased HRV does indeed require further investigation. Furthermore, an experimental study has revealed that intracerebroventricular injections of IL‐6 do not result in a decrease in HRV in mice (Hajiasgharzadeh et al., 2011), which suggests that the direct interaction of IL‐6 within brain centers (e.g., cardiovascular regulatory centers in the medulla) is not the major mechanistic basis by which it confers decreased HRV. However, this does not negate the fact that decreased HRV may in part be conferred due to uncoupling exhibited between the central brainstem and the SAN of the heart via different mechanisms which are yet to be delineated. Indeed, defective CNS cardiovascular control has been reported in rats with cirrhosis (Song et al., 2001), as altered patterns of Fos staining have been documented in the brainstem, especially in cardiovascular control centers such as the ventrolateral medulla and the nucleus of the solitary tract (Song et al., 2001). Thus, reinforcing the need to mechanistically determine the potential effect of such defective brainstem control on patients’ HRV. Withal, these studies collectively highlight that systemic inflammation conferred by IL‐6 is likely a significant contributory factor conferring decreased HRV (Bhogal et al., 2019; Haddadian et al., 2013; Mani et al., 2009), however the exact mechanistic basis by which IL‐6 elicits this is yet to be elucidated and requires further investigation.

Impaired neuropsychiatric function is common in patients with cirrhosis and is known as hepatic encephalopathy (HE). Interestingly, reduced HRV is reported to be highly correlated with the severity of HE in patients with cirrhosis (Mani et al., 2009). Decreased HRV has also been documented in other conditions characterized by neuropsychiatric impairment, namely dementia (Kim et al., 2006; Zulli et al., 2005) and in depression (Vigo et al., 2004). Exceedingly, HE is a well‐documented manifestation of decompensated cirrhosis, whereby pro‐inflammatory cytokines have been reported to be pivotal in its pathogenesis (Jover et al., 2006a, 2006b; O'Beirne et al., 2006; Shawcross et al., 2004), such that their concentration increases in parallel with the severity of HE (Genesca et al., 1999; Odeh et al., 2004; Odeah et al., 2005), as well as in other neuropsychiatric conditions (Anisman & Merali, 2002; Capurun et al., 2008; Gimeno et al., 2008; Henry et al., 2008; Vaccarino et al., 2008). Moreover, given that the reduction in HRV in patients with cirrhosis has been correlated with increased cytokine levels (Genesca et al., 1999; Tilg et al., 1992), namely IL‐6 (Aronson et al., 2001; González‐Clemente et al., 2007; Tateishi et al., 2007), this highlights the central role systemic inflammation potentially elicits in giving rise to this manifestation and in precipitating reduced HRV. Importantly, this is reinforced by the study by Mani et al., revealing SD2, a long term HRV index to be substantially decreased and also significantly correlated to the severity of HE and thus the extent of neuropsychiatric impairment, independent to the severity of liver dysfunction. Furthermore, the presence of HE as well as the decreased HRV exhibited, both confer poor prognosis in patients with cirrhosis (Bustamante et al., 1999; Fleisher et al., 2000). Crucially, Mani et al., revealed that a 1 millisecond decrease in SD2 was associated with a significant increase in the relative risk of mortality, specifically, a 7.7% increase. In addition, increasing IL‐6 concentrations were found to be significantly correlated with both parameters, HE and the reduction in SD2, substantiating previous evidence highlighting its pathogenic role (Jover, et al., 2006a, 2006b; O’Beirne et al., 2006; Shawcross et al., 2004). Hence, pro‐inflammatory cytokine induced neural dysfunction may indeed be central in causing both the neuropsychiatric impairment exhibited in HE and decreased HRV, by affecting specific regions of the brain involved in both cognitive function and in cardiovascular regulation, namely the cortex and subcortical cardiovascular medullary control centers, respectively (Dantzer et al., 2008). Importantly, the latter has indeed been found to be impaired in animal models of cirrhosis (Song et al., 2001). Thus, corroborating previous evidence revealing that cognitive impairment exhibited in patients with primary biliary cirrhosis is indeed independent to the extent of hepatic dysfunction and is primarily correlated with the extent of defective autonomic control (Newton et al., 2008). Moreover, as many features of long term HRV are related to higher brain function, as well as areas involved in hypothalamic thermoregulatory neural regulation, whether HE, decreased HRV and impaired thermoregulation indices are mechanistically related is an area of further investigation (Garrido et al. 2017a, 2017b).

An area of controversy remains as to whether decreased HRV is as a result of defective cardiomyocyte responsiveness, this is since cytokines have been found to diminish beta‐ adrenergic signaling, resulting in pronounced adrenergic hypo‐responsiveness (Gaskari et al., 2006; Prabhu, 2004), known as cirrhotic cardiomyopathy. Cirrhotic cardiomyopathy is indeed a frequent complication exhibited in cirrhosis, resulting in defective contractile activity of the myocardium in response to physiological stress and stimulation (Myers & Lee, 2000), however, is nonetheless difficult to diagnose due to the profound systemic vasodilation exhibited in cirrhosis, concealing the presence of cardiomyopathy (Liu et al., 2017, 2022). Several mechanistic theories have indeed been proposed such as abnormalities in the cardiomyocyte membrane, as well as an increased biosynthesis of nitric oxide (NO), and other mediators such as carbon monoxide and endocannabinoids eliciting a role in its pathogenesis (Gaskari et al., 2005; Yoon et al., 2020), Thus, given that NO has been found to be pivotal in the pathogenesis of the impaired cardiac adrenergic response exhibited in cirrhosis, whether this manifestation contributes to the reduction of HRV in patients, is yet to be more accurately ascertained. However, experimental studies conducted to further understand the mechanistic basis of cirrhotic cardiomyopathy have revealed that pharmacological interventions that restore cirrhotic cardiomyopathy do not correct HRV in experimental cirrhosis (Mani, Ollosson, et al., 2006; Mani, Ippolito, et al., 2006). Notably, research in animal models is indeed suggestive of the fact that cirrhotic cardiomyopathy and reduced HRV may indeed have different etiologies, as NO synthase inhibitors or N‐acetylcysteine which are known to ameliorate cirrhotic cardiomyopathy had no effect on reduced HRV (Mani, Ollosson, et al., 2006; Mani, Ippolito, et al., 2006). Thus, reinforcing the notion that reduced HRV may not be reflective of cirrhotic cardiomyopathy exhibited in cirrhosis.

Crucially, studies have revealed that despite HRV being already reduced in cirrhosis, a further inflammatory insult, namely acute endotoxin challenge in cirrhotic rats does not result in a further reduction in HRV, which was nonetheless exhibited in the non‐cirrhotic group (Haddadian et al., 2013). From a mechanistic point of view, endotoxin challenge in non‐cirrhotic rats led to an impaired cardiac pacemaker response and thus decreased chronotropic responsiveness to cholinergic stimulation. Thus, highlighting how uncoupling may have occurred between afferent vagal innervation and the SAN, a phenomenon which was lost in cirrhotic rats. Therefore, revealing that cirrhotic rats develop tolerance to the effects of bacterial endotoxin, which is postulated to occur at the level of the SAN (Haddadian et al., 2013). This is mechanistically important as this suggests that the reason for reduced HRV in cirrhosis might be different from acute inflammatory disorders, such as sepsis and acute myocardial infarction (MI). This is reinforced by the fact that clinical studies have also shown that HRV indices that predict mortality in cirrhosis, are distinct from those used in acute disorders such as MI whereby, short‐term HRV indices are good predictors of poor prognosis in patients with MI, whereas in cirrhosis, long‐term variability indices are better independent predictors (Oyelade et al., 2020).

**FIGURE 2:**
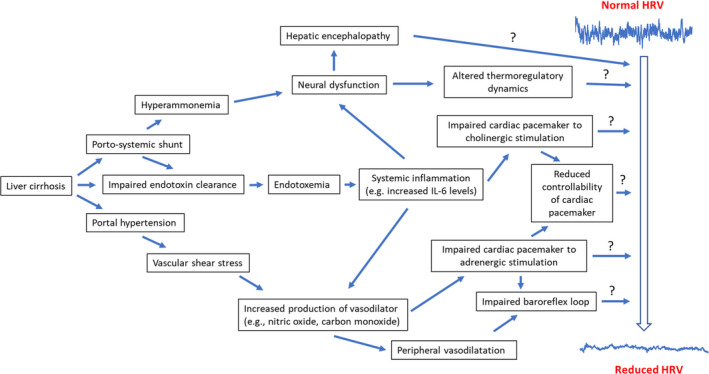
A schematic diagram on mechanisms that may lead to reduced heart rate variability (HRV) in cirrhosis

In essence, decreased HRV in patients with cirrhosis is indeed reflective of a network of factors exhibiting dysfunction (Figure 2), such that the reduction is likely a manifestation of a decrease in many aspects of cardiovascular control, which elicit their regulatory effects over specific time points (Altimiras, 1999; Goldberger et al., 2002). However, the exact mechanistic basis of decreased HRV in patients with cirrhosis is yet to be ascertained more definitively. Indisputably, systemic inflammation is presumptively a central mechanism in conferring decreased HRV, however further research is nonetheless required to elucidate a more definitive link, highlighting the need for a network physiology approach.

## 3 PROGNOSTIC IMPLICATIONS

Pre‐eminently, several lines of evidence have showcased the fact that decreased HRV is able to independently predict survival of patients with cirrhosis (Bhogal et al., 2019; Bottaro et al., 2020; Mani et al., 2009). Although, various indices such as fractal and entropy analysis have been used to analyze HRV (Bian et al., 2012;Costa et al., 2002; Jiang et al., 2013; Peng et al., 1995; Satti et al., 2019; Song et al., 2013; Zheng et al., 2010)ndices from the Poincaré plot have been documented to possess greater prognostic capability in cirrhosis (Bhogal et al., 2019). Specifically, Bhogal et al., revealed that SD2 and cSDNN (SDNN corrected for heart rate) can predict the 18‐month survival of cirrhotic patients independently to MELD and thus the extent of liver dysfunction from 10‐min ECG recordings (for more information about details of HRV measures see Appendix 1 and Figure 1). These indices were found to exhibit prognostic capacity independent to the effect of age, and gender as well as the cause of their cirrhosis and treatment with beta blockers. Such prognostic capacity is reflective of defective cardiovascular regulatory mechanisms, such as autonomic control. However, further research in the future is needed to correlate these indices with the cause of death, alongside measuring the severity of other manifestations such as the extent of cirrhotic cardiomyopathy in the patient cohort, as well as portal hypertension (Bhogal et al., 2019). Nonetheless, indices from the Poincare’ plot have indeed transformed the landscape of prognostication in patients with cirrhosis. Crucially, Satti et al., (2019) extended the Poincare’ plot analysis, by calculating the correlation between sequential data points in a time‐series, rather than between two consecutive points. In contrast, the traditional Poincare’ plot only calculates the correlation between two distinct points in a time series. Thus, this ‘extended’ Poincare plot depicts the extent of autocorrelation exhibited between present and past physiological time points and hence memory possessed within patients’ HRV (Shirazi et al., 2013). Importantly, Satti et al., (2019) revealed that decompensated patients exhibited a greater degree of autocorrelation within their time‐series in comparison to patients with less severe diseases, reflective of increased memory within their HRV. Such a physiological system is postulated to be less controllable and thus more challenging to regulate (Ghafari et al., 2017; Mazloom et al., 2014; Shirazi et al., 2013) as well as being less able to adapt to physiological challenges, as a result of such defective homeostasis. Indeed, extended‐SD2 reflective of long‐term variability and autocorrelation in patients’ HRV analysis was found to predict survival independently to MELD, currently used in the clinic to assess and stage the severity of liver cirrhosis and thus prognosticate patients for liver transplantation. Thus, highlighting that it holds the potential in being employed as a non‐invasive prognostic indicator, and can greatly increase the likelihood of selecting those individuals most in need for the liver transplantation in conjunction with MELD.

The exact mechanistic basis by which long term HRV can elucidate poor prognosis is unknown, however extended SD2 and thus patients’ long term HRV is suggested to be influenced by the baroreflex loop, as well as hypothalamic thermoregulatory control mechanisms. Indeed, initial reports were suggestive of the notion that defective thermoregulation in cirrhosis (Garrido et al., 2017a, 2017b; Mani et al., 2018) may be associated with long term HRV (Satti et al., 2019). Moreover, other indices of long term HRV such as VLF (very low frequency) have also been reported to be modulated by core body temperature (CBT) fluctuations (Akselrod et al., 1981; Fleisher et al., 1996). Thus, reinforcing the hypothesis that impaired thermoregulatory function may be conferring the reduction in long term HRV, especially in SD2, a better index for long term HRV quantification, as VLF is in fact subject to influence by the computational analysis (de‐trending) conducted on the ECG recordings. Indeed, this impairment has been reported in CBT and peripheral skin temperature profiles of patients with cirrhosis (Garrido et al., 2017a, 2017b). Remarkably, Bottaro et al., (2020) revealed that the fluctuations in patients’ temperature‐time series holds key prognostic information. Specifically, reduced short‐term temperature variability, and its fractal‐like fluctuations could predict the 12‐month survival of patients with cirrhosis from a 24‐h temperature recording, independent to MELD and thus the extent of disease severity. However, the prognostic ability of long term decreased HRV and decreased short‐term temperature variability in predicting survival were found to be independent of each other (Bottaro et al., 2020). Nonetheless, since both temperature variability and HRV are heavily influenced by the autonomic activity, their independence reveals that HRV, and body temperature variability indices may indeed assess the integrity of autonomic networks from different complementary angles.

HRV indices such as SDNN, reflecting the standard deviation of patients’ R‐R intervals have also been found to be significantly decreased and also independently predict 3‐month survival in patients with cirrhosis (Jansen et al., 2019). Repeated measures were also found to be associated with the extent of disease severity and thus decompensation, whereby substantially lower levels were documented in patients who progressed to acute on chronic liver failure (ACLF), in comparison to those solely exhibiting decompensation. In the latter instance, these patients nonetheless exhibited lower SDNN values in comparison to outpatients with stable disease. Crucially, SDNN levels were negatively correlated with an increased CLIF‐CAD score, a powerful predictor of ACLF and survival in decompensated patients (Jalan et al., 2015; Jansen et al., 2019). Moreover, decreased SDNN levels were found to be significantly inversely correlated with markers of systemic inflammation, pathognomonic of ACLF, such as the leukocyte count and concentration of serum C‐reactive protein (CRP), as well as increased IL‐6 levels. Indeed, in patients with cirrhosis, such systemic inflammation is associated with poor prognosis, whereby decompensated patients exhibit increased leukocyte counts and CRP concentration concomitant with more progressive disease (Jalan et al., 2015; Piano, Morando, et al., 2017; Piano, Tonon, et al., 2017). Notably, in this study 50% of patients’ cause of death was due to infection. Thus, reinforcing the central role systemic inflammation elicits in conferring reduced HRV, which is significant since cirrhotic patients exhibit a substantially increased susceptibility to developing infections which are known triggers of ACLF. Importantly, this has been reported in approximately 50% of patients in developed countries in the west. Furthermore, once patients progress to ACLF their susceptibility to life‐threatening infections is further exacerbated, which often prove fatal. Hence, the decrease in HRV may indeed manifest as a consequence of inflammation which is prominent in patients with ACLF (Clària et al., 2016; Hernaez et al., 2017). This corroborates with other research in the field of HRV analysis, whereby a significant negative correlation is exhibited between CRP and decreased SDNN in the context of unstable angina pectoris (Lanza et al., 2006). Furthermore, in patients with sepsis, decreased SDNN levels are also synonymous with poor prognosis (de Castilho et al., 2017), whereby patients in ICU exhibiting critically low SDNN levels had a significantly higher chance of mortality. Interestingly, in the study by Jansen et al., (2019) throughout the course of the follow up period, patients’ whose SDNN levels increased were found to exhibit a greater chance of disease amelioration, in contrast to those whose SDNN decreased for whom the prognosis was worse. Thus, highlighting how repeated SDNN quantification over the course of a patient’s disease will indeed enable a more accurate prediction of the likely course and therefore prognosis a patient exhibits and crucially whether they exhibit a positive response to treatment. Moreover, given that leukocyte counts, and CRP levels did not offer prognostic information, remote SDNN quantification can also allow for timely clinical intervention such as antibiotics to prevent progression to ACLF and thus decompensated disease characterized by systemic inflammation, to ultimately prevent organ failure from precipitating. Nonetheless, future studies are indeed needed, involving repeated measures accrued over a longer follow up period, to more accurately validate the predictive power of SDNN quantification. Furthermore, recent studies show that SDNN is affected by the mean heart rate, thus revealing that it is best to correct SDNN measures for the assessment of autonomic function (Monfredi et al., 2014). Bhogal et al., (2017) also showed that the correction of SDNN for basal heart rate using a formula proposed by Monfredi et al., makes SDNN a better index for prognostication in patients with cirrhosis. This slight modification can also be added to improve the prognostic value of SDNN in the prediction of clinical deterioration.

Most HRV indices can be used for the assessment of autonomic function in individuals with normal sinus rhythm. However, many patients may exhibit common arrhythmias such as a benign premature ventricular complex (PVC). Thus, heart rate turbulence (HRT) represents an alternative index reflective of the functionality of the autonomic nervous system, whereby the heart rate is changed in a biphasic manner in response to a PVC (Oyelade et al., 2020). Importantly, PVCs have been documented in the vast majority of patients with cirrhosis, within their 24 h ECGs and is also an expected phenomenon (Kennedy et al., 1985), such that HRT is frequently exhibited post PVCs. Crucially, Jansen et al., (2018) revealed indices of HRT, such as turbulence onset to correlate directly with disease severity and thus greater Child‐Pugh and MELD scores, whereby a greater proportion of patients progressed to decompensation within a six‐month period. Furthermore, turbulence onset could predict 12‐month survival independent to the Child‐Pugh and MELD score (Oyelade et al., 2020).

Withal, these findings showcase that HRT is indeed a valid alternative to long term HRV indices in patients exhibiting an atypical sinus rhythm and thus PVCs. However, atrial fibrillation, a common arrythmia documented in the elderly, remains a major challenge, preventing HRV and HRT indices from accurately assessing autonomic function in these subsets of patients. Thus, further research is needed for the development of novel methods for non‐invasively assessing the autonomic nervous system in patients with common arrythmias such as atrial fibrillation.

### 3.1 HRV and liver transplantation

The prediction of survival is important in cirrhosis and helps clinicians in allocating liver transplants to those patients most in need. Since HRV analysis holds invaluable prognostic information for patients with cirrhosis, it has been suggested that HRV analysis may be added to current prognostic scores (such as MELD) in order to increase the accuracy of organ allocation. On this note, Chan et al., (2017) reported that HRV indices (i.e., deceleration capacity and heart rate complexity) are highly accurate in predicting 1‐year post‐transplantation mortality. Their concomitant use with the MELD score increased the overall accuracy in predicting mortality after liver transplantation from 0.752 to 0.912. Although this study indicates a powerful prognostic value for HRV indices post‐transplantation in cirrhosis, it is however not easy to conclude that HRV analysis can be used as a novel index for organ allocation in patients awaiting liver transplantation. As alluded to by Bhogal et al., (2019), prediction of post‐transplant mortality might indicate that reduced HRV is in essence a co‐morbidity that increases the chance of death post‐transplant, rather than a physiological index that is improved by liver transplantation. This is further reinforced by the fact that parasympathetic impairment in cirrhosis (measured by short‐term HRV indices) is not affected or even improved in the long‐term after transplantation (Baratta et al., 2010), despite improvements in liver, neural and renal function. Thus, given that those exhibiting decreased HRV have a poorer prognosis post‐transplant, it can indeed be utilized as a co‐morbidity factor, in a similar fashion to other co‐morbidities, such as non‐hepatic cancers when prioritizing patients for liver transplantation (Bhogal et al., 2019). With regards to autonomic function, as reduced heart rate complexity did not improve or exhibit any significant changes post‐transplant, this highlights that cardiac autonomic control did not significantly improve 1 year post transplantation (Chan et al., 2017). This is in line with other investigations reporting a temporary decline in autonomic function within the first 6 months post‐transplant, however this was nonetheless followed by a gradual improvement in measures of cardiac autonomic function (Bhatti et al., 2021). Thus, whether the inclusion of HRV indices (or other autonomic measures) during liver transplant allocation improves post‐transplant survival is yet to be delineated. Ultimately, this will help ascertain whether reduced HRV negatively affects transplantation outcome (as a co‐morbidity) or whether it can indeed assist in allocating the organ to the recipient most in need.

## 4 FUTURE IMPLICATIONS

On whole, decreased HRV in cirrhosis may be an indicator of physiological network disruption in cirrhosis (Figure 2). Furthermore, monitoring HRV indices in patients with cirrhosis has the potential to help clinicians in making clinical decisions and also introducing timely clinical interventions to prevent any further deterioration from manifesting, which would inevitably confer a huge cost on healthcare services. Thus, in the future indices from the Poincare’ plot may assist in identifying those in critical need of transplantation or indeed hospital admission and also potentially aid in predicting post‐procedure mortality, which nonetheless requires further investigation. Moreover, their non‐invasive and inexpensive nature, coupled with the fact that only a single 10‐min ECG recording, with the use of a single computational script programmed into bedside monitors, can predict the 12‐month survival of cirrhotic patients, would provide a substantial ‘health economic benefit’. Hence, highlighting the immense potential physio‐markers hold, which is in line with previous research revealing other physio‐markers such as EEG in conjunction with MELD to increase the accuracy of prognostication (Montagnese et al., 2015).

Future studies are nonetheless needed to determine measures to correct the reduction exhibited in patients’ HRV and concurrently validate mechanistic links such as that exhibited between inflammation and reduced HRV in cirrhosis. Since IL‐6 inherently elicits a central role in propagating systemic inflammation in cirrhosis and is indeed substantially correlated to decreased HRV indices, whether treatment with tocilizumab, a monoclonal antibody targeting IL‐6, can restore decreased HRV to its normal range is an area of further investigation.

Likewise, substantial evidence has been reported for the use of exercise in increasing HRV. Thus, whether specific regimes confer more beneficial effects on HRV concomitant with being appropriate for patients exhibiting decompensated disease is yet to be ascertained and formulated for this patient population.

However, recent reports have revealed more robust therapeutic strategies that have the potential to restore HRV in experimental cirrhosis. Notably, experimental studies have revealed that alteration of the pattern of respiration via fractal‐like mechanical ventilation, in a rat model of ACLF post endotoxin injection, has been associated with increased oxygen saturations and prevented the reduction in HRV, specifically in short‐term HRV indices (Nataj et al., 2018). This was concomitantly documented with decreased liver injury and importantly a decrease in death rate. Mechanistically, fractal‐like mechanical ventilation has been documented to augment respiratory sinus arrythmia (RSA) (Mutch et al., 2005) which is indeed quantified by short‐term HRV indices. Importantly, respiratory sinus arrhythmia is mediated by vagus nerve activity (Katona & Jih, 1975) which in itself elicits a key role in combatting multi‐organ failure in the context of systemic inflammation (Rosas‐Ballina et al., 2011; Tracey, 2007). This corroborates with the critical role the vagus nerve elicits in the anti‐inflammatory vagal pathway, whereby vagal innervation activating T cells expressing choline acetyltransferase (Rosas‐Ballina et al., 2011), results in the subsequent release of acetylcholine, which acts on α7 nicotinic acetylcholine receptors expressed on macrophages. Crucially, this has been suggested to inhibit the production of pro‐inflammatory cytokine generation as an attempt to dampen systemic inflammation (Mazloom et al., 2013; Rosas‐Ballina et al., 2011; Tracey, 2007). Furthermore, hepatocytes have been found to express alpha‐7 nicotinic acetylcholine receptors (Hajiasgharzadeh et al., 2014) and CNS innervation has also been found to alter the extent of liver damage exhibited (Eftekhari et al., 2014). Thus, highlighting that fractal‐like mechanical ventilation could potentially be therapeutically exploited in the future to treat patients with acute on chronic liver failure who require ventilatory support. These studies also showcase the immense potential vagal nerve stimulation could have in treating conditions characterized by systemic inflammation which often result in multi‐organ failure (Eftekhari et al., 2014; Hajiasgharzadeh & Baradaran, 2017). However, this nonetheless requires further research to validate, especially in patients with cirrhosis who have progressed onto develop ACLF. In addition, it has also been postulated that decreased HRV may be indicative of decreased activity in the vagal anti‐inflammatory pathway and due to a greater extent of baseline systemic inflammation, results in organ failure (Hajiasgharzadeh & Baradaran, 2017; Hajiasgharzadeh et al., 2014; Mazloom et al., 2013) which may potentially be exacerbated in response to an acute infection. Thus, this may increase the susceptibility of sepsis in patients with cirrhosis which is evidently a very pertinent concern in the clinic. However, the exact extent to which this anti‐inflammatory pathway elicits its effect in dampening inflammation in the context of cirrhosis is yet to be ascertained. Ultimately, elucidating this could pave way to therapies such as non‐invasive vagal stimulation to be used in the clinic, which may remarkably hold the potential to revolutionize treatment and survival rates for patients with cirrhosis.

In the future, studying the interaction between a network of organs in the context of systemic inflammation will help determine the exact extent to which specific organs contribute towards decreased HRV (Asada et al., 2016; Bartsch et al., 2015; Ivanov et al., 2016; Xiong et al., 2017). Subsequently, enabling us to ascertain the exact mechanistic basis by which reduced HRV is indicative of poor prognosis. Shashikumar et al., (2017) have indeed suggested that delineating the interaction of organs, specifically those of the cardiovascular and respiratory systems using network analysis, will help in understanding the exact extent to which this interaction results in poor survival rates in septic patients. Thus, highlighting the inherent need for a multi‐scale network construction analysis, which will ultimately enable clinicians to both comprehend and target multiple determinants, as well as impaired cardiovascular control mechanisms to therapeutically combat decreased HRV in critically ill patients.

To conclude, HRV analysis has indeed transformed the landscape of assessing prognosis in patients with cirrhosis. Crucially, in the future HRV analysis may indeed increase the accuracy of current prognostic factors and be used in conjunction with other physio‐markers such as EEG and body temperature variability analysis, to ultimately increase the number of lives saved.

## CONFLICT OF INTEREST

The authors declare that the review was written in the absence of any commercial or financial relationships that could be construed as a potential conflict of interest.

## AUTHOR CONTRIBUTIONS

NUHA and ARM contributed to the conceptualization. NUHA contributed to the writing of the original draft. ARM contributed to the writing – review and editing. All authors contributed to the article and approved the submitted version.

## RECORDING

Any physiological signal that provides information about the cardiac rhythm with high resolution can be used for recording. For example, electrocardiograms (ECG), photoplethysmograms and piezoelectric pulse sensors are frequently applied for assessing the cardiac rhythm and HRV measurement. Furthermore, ECG data can be also extracted from electro encephalogram (EEG) recordings, frequently used for assessing hepatic encephalopathy in the context of cirrhosis (Mani et al., 2009).

The sampling rate for recording the cardiac signals should be higher than 128 Hz. The duration of recording varies in different studies, from 5 min to 24 h. However, a minimum of 5 min is recommended for calculating the most common indices of HRV and still provides prognostic information. A detailed discussion around the basic recording requirements of assessing HRV is highlighted further by the Task Force of The European Society of Cardiology and The North American Society of Pacing and Electrophysiology (1996).

## EXTRACTION OF BEAT‐TO‐BEAT INTERVAL TIME‐SERIES

Prior to HRV analysis, beat‐to‐beat intervals are required to be extracted from raw cardiac signals (e.g., R‐R intervals from ECGs). This cannot be carried out manually and is quantified using computational algorithms. There are several open‐source softwares for this extraction (e.g., Pichot et al., 2016; Tarvainen et al., 2014).

## HRV ANALYSIS

### C.1 Time‐domain analysis

Total HRV can be measured by simply measuring the standard deviation of the inter‐beat intervals. Commonly, this index is known as **SDNN** (**S**tandard **D**eviation of **N**‐**N** interval, which in the case of an ECG can be the R‐R interval). Since SDNN is influence by the basal heart rate, total HRV is hence corrected for the basal heart rate giving rise to the corrected SDNN index (**cSDNN**) which is then use for further analysis. The most optimal formula for this correction is (Monfredi et al., 2014):

$$cSDNN=\frac{\mathrm{SDNN}}{e^{\frac{‐Heartrate}{58.8}}}$$

Furthermore, there are other time‐domain analyses for HRV such as SDANN (Standard deviation of the averages of N‐N intervals in all 5 min segment) and RMSSD (the square root of the mean of the sum of the squares of differences between adjacent N‐N intervals). For further information please see Shaffer & Ginsberg, 2017.

### C.2 Measurement of short‐term and long‐term HRV

Mechanistically and for prognostic reasons, HRV is often categorized into short and long‐term components which short‐term and long‐term HRV indices aim to measure. Several analytical methods can be used for this purpose, including spectral analysis and the Poincare’ plot.

#### C.2.1 Spectral analysis

This method aims to decompose HRV into its constituent frequencies. Analytically, Fast Fourier Transform or Autoregression is used for the spectral analysis. A typical spectrogram of HRV depicts high frequency (LF), low frequency (LF) and very low frequency (VLF) bands as shown in Figure 1.

#### C.2.2 Poincare’ plot

This method is a graphical representation of HRV, whereby it aims to illustrate the correlation between adjacent points within the inter‐beat interval time‐series (Figure 1). A typical Poincare’ plot is elliptical in shape, in which the diameters reflect short‐term and long‐term HRV components (Figure 1).

### C.3 Non‐linear methods

The fluctuations exhibited within the cardiac sinus rhythm are complex and do not follow a typical linear pattern. Thus, non‐linear methods aim to quantify this complexity. Information in complex systems can be measured using *entropy*, which quantifies the degree of regularity possessed within the time‐series. Hence, entropy indices such as Sample Entropy (SampEn), Approximate Entropy (ApEn) and Multiscale Entropy are used for non‐linear analysis (Bhogal et al., 2019).

Furthermore, the cardiac rhythm exhibits a fractal‐like pattern which reflects the degree of self‐similarity it possesses at different scales at which it is measured (Goldberger et al., 2002). **D**e‐trended **F**luctuation **A**nalysis (DFA) thus aims to mathematically “de‐trend” the general R‐R interval time‐series at different scales to reveal its fractal pattern. This is then analyzed further to reveal differences in its structure in the context of disease such as cirrhosis.

## OPEN‐SOURCE SOFTWARE AVAILABLE FOR HRV ANALYSIS

The following open‐source softwares are available for both linear and non‐linear methods of HRV analysis:

Kubios HRV: Tarvainen et al., 2014

HRVanalysis: Pichot et al., 2016

CEPS: Mayor et al., 2021

## APPENDIX 1 BASIC REQUIREMENTS FOR MEASUREMENT OF HEART RATE VARIABILITY (HRV) IN CLINICAL PRACTICE

There are several indices used to quantify HRV measurement. Extensive reviews are available for further information regarding each specific index (see Shaffer & Ginsberg, 2017). This appendix outlines the key principles underpinning the measurement of HRV relevant to predicting survival in cirrhosis.
